# Lanthanide (Eu, Tb, La)-Doped ZnO Nanoparticles Synthesized Using Whey as an Eco-Friendly Chelating Agent

**DOI:** 10.3390/nano12132265

**Published:** 2022-06-30

**Authors:** Carolina Picasso, Yolanda Salinas, Oliver Brüggemann, Markus Clark Scharber, Niyazi Serdar Sariciftci, Olavo D. F. Cardozo, Eriverton S. Rodrigues, Marcelo S. Silva, Andreas Stingl, Patricia M. A. Farias

**Affiliations:** 1 Institute of Inorganic Chemistry, Johannes Kepler University Linz, Altenberger Straße 69, 4040 Linz, Austria; carolina.picasso@jku.at; 2 Institute of Polymer Chemistry (ICP), Johannes Kepler University Linz, Altenberger Straße 69, 4040 Linz, Austria; oliver.brueggemann@jku.at; 3 Linz Institute for Organic Solar Cells (LIOS), Physical Chemistry Institute, Johannes Kepler University Linz, Altenberger Straße 69, 4040 Linz, Austria; markus_clark.scharber@jku.at (M.C.S.); serdar.sariciftci@jku.at (N.S.S.); 4 Post-Graduate Program on Electrical Engineering, Federal University of Pernambuco, Cidade Universitaria, Recife 50670-901, Brazil; olavo.cardozo@ufpe.br; 5 Phornano Holding GmbH, Kleinengersdorferstrasse 24, 2100 Korneuburg, Austria; andreas.stingl@phornano.com; 6 Post-Graduate Program on Material Sciences, Federal University of Pernambuco, Cidade Universitaria, Recife 50670-901, Brazil; eriverton.rodrigues@if-sertao-pe.edu.br; 7 Federal Institute of Education, Science and Technology of Sertão Pernambucano, Salgueiro 56000-000, Brazil; marcelo.silva@ifsertao-pe.edu.br

**Keywords:** zinc oxide nanoparticles, doped nanomaterials, eco-friendly fluorescent nanomaterials, safe and sustainability in semiconductor nanomaterials

## Abstract

Strategies for production and use of nanomaterials have rapidly moved towards safety and sustainability. Beyond these requirements, the novel routes must prove to be able to preserve and even improve the performance of the resulting nanomaterials. Increasing demand of high-performance nanomaterials is mostly related to electronic components, solar energy harvesting devices, pharmaceutical industries, biosensors, and photocatalysis. Among nanomaterials, Zinc oxide (ZnO) is of special interest, mainly due to its environmental compatibility and vast myriad of possibilities related to the tuning and the enhancement of ZnO properties. Doping plays a crucial role in this scenario. In this work we report and discuss the properties of undoped ZnO as well as lanthanide (Eu, Tb, and La)-doped ZnO nanoparticles obtained by using whey, a by-product of milk processing, as a chelating agent, without using citrate nor any other chelators. The route showed to be very effective and feasible for the affordable large-scale production of both pristine and doped ZnO nanoparticles in powder form.

## 1. Introduction

Nanotechnology has often been listed among leading technologies towards sustainable growth and development [1,2,3,4,5,6]. Green or sustainable nanomaterials’ concepts are based on sustainable green chemistry principles through the design, production, usage, and the entire life cycle of nanomaterials. The development of synthetic routes that enable the production of nanomaterials throughout the use of safer and renewable reagents and solvents is also a remarkable need in this scenario [4]. Semiconductors account for a large part of the annual demand worldwide for nanomaterials. Among them, ZnO nanoparticles (NPs) are the most largely produced and studied [7,8,9].

ZnO is a II–VI semiconductor, with a wide band gap and large exciton binding energy (60 meV at room temperature), that presents high thermal and chemical stability, hence used in a vast myriad of applications. Over the years a wide variety of chemical, physical and hybrid methods for the synthesis of safer and more sustainable production of ZnO NPs has been reported [10,11,12,13,14]. However, these approaches still present some significant setbacks, such as the usage of highly toxic and hazardous chemicals, such as ethylene glycol, among others. In contrast, a previously reported citric-acid based sol-gel method [15] for the synthesis of doped and undoped ZnO NPs uses whey to promote citrate polymerization [10,11,12,13,14,15].

Whey is a liquid by-product of cheesemaking and is mainly composed of fats, globular proteins, lactose, and minerals. During the process of cheese production, after milk coagulation only whey remains. Proteins present in whey are acid-soluble and correspond to approximately 20% of the total protein content in bovine milk. Heat treatment of whey protein is a typical industrial technique that results in structural changes in proteins [16,17].

Controlled denaturation of the native globular proteins in whey can be achieved by controlling the heating and gives rise to a three-dimensional network [17]. This feature is quite similar to the above-mentioned ability of citric acid in sol-gel methods and illustrates the potential of using whey, without any additional reagent, to chelate metallic ions and to produce a three-dimensional network that acts as a matrix in which nanosized metal-oxide crystals can nucleate. Undoped and lanthanide (Eu, Tb, and La)-doped ZnO NPs were obtained by this method, with a great potential to be a very effective and feasible method for large-scale production of both pristine and doped ZnO NPs.

Cadmium Telluride (CdTe) and Cadmium Selenide (CdSe) are also II–VI semiconductors, of nanometric size range and largely used in similar applications to ZnO. However, health and environmental aspects have pointed out significant concerns related to their production and their use. Doping ZnO NPs with rare-earth elements opens a wide diversity of alternatives to Cd-based quantum dots due to their advantages in terms of safety, sustainability, and environmental compatibility. This work focusses on developing a safe and sustainable route for the scalable production of nanomaterials, maintaining their tunable optical properties, opening new roads to eco-friendly and fluorescent semiconductor nanomaterials.

## 2. Materials and Methods

### 2.1. Chemicals

For the present work, the following reagents were used to produce undoped ZnO NPs and rare-earth (Eu, Tb, and La) doped ZnO NPs: ultra-pure water (Milli Q, Type I), hexa-hydrated zinc nitrate (Zn(NO_3_)_2_ · 6H_2_O) 99.9%, hexa-hydrated europium nitrate (Eu(NO_3_)_3_ · 6H_2_O) 99.99%, hexa-hydrated terbium nitrate (Tb(NO_3_)_3_ · 6H_2_O) 99.99% and hexa-hydrated lanthanum nitrate (La(NO₃)₃ · 6H₂O) 99.99%. As a by-product of their curd production, whey was sourced from local farmers in Lower Austria and used without additional purification. Except by whey, all chemicals were purchased from Merck KGaA, Darmstadt, Germany and used without additional purification unless otherwise specified.

### 2.2. Synthesis of ZnO Doped with Eu, Tb, and La

Zinc Oxide (ZnO) undoped NPs were obtained in powder form by modifying a sol-gel technique reported in a previous work [15]. In the present work, bovine milk whey was added to an aqueous solutions of Zn(NO_3_)_2_ · 6H_2_O under heating at 80 °C, without the addition of citric acid or any other chelating agent. To produce lanthanide (Ln = Eu, Tb, and La)-doped ZnO NPs, the respective lanthanide precursor was added (Ln(NO_3_)_3_ · 6H_2_O, Ln = Eu, Tb, and La) into the solution containing Zn(NO_3_)_2_ · 6H_2_O in a ratio of 5% (mass/mass), after adding whey to the heated solution, for each case of doped ZnO. Each mixture was separately kept under heating (80 °C) and stirred for 1 h. Stable xerogels were formed for each Ln-doped ZnO and subsequently calcined at 400 °C for 1 h. As a result, the following nanosized powders were obtained as pristine ZnO, Europium-doped ZnO (ZnO:Eu), Terbium-doped ZnO (ZnO:Tb), and Lanthanum-doped ZnO (ZnO:La) and characterized as follows.

### 2.3. TEM-EDS Analysis

Samples for transmission electron microscopy and energy disperse spectroscopy (TEM-EDS analysis) were prepared by drop casting an ethanol dispersion of each sample onto holey-carbon TEM copper grids. Detailed structural characterization of the samples was obtained using a JEOL JEM-2200FS (JEOL Ltd., Peabody, MA, USA) transmission electron microscope (TEM) operated at 200 kV. The size and shape distributions were evaluated by analysis of TEM micrographs using ImageJ software (v.1.53e, National Institute of Health, Bethesda, MD, USA) and 500 particles for each sample. Particle size histograms were fitted with a Gaussian distribution function. The shape distribution was quantified using the aspect ratio factor (maximum Feret diameter/minimum Feret diameter) typical for the description of spheroids (rods). Quantitative EDS microanalysis was performed using an X-Max^N^ 80 T Silicon Drift Detector (Oxford Instruments, Abingdon, UK). Data collection and analysis were realized via the AZtec integrated analysis system (Oxford Instruments, Abingdon, UK). EDS data acquisition was performed at an accelerating voltage of 200 kV, fixed channel width of 10 eV (2048 channels and 20 keV energy range), and by simultaneously applying pulse pile up and absorption corrections. Quantitative EDS microanalysis was realized based on the Cliff-Lorimer method using theoretical *k*-factors incorporated in Aztec v4.3 [18,19]. Copper was used as the calibration element. EDS Spectrum Images (SIs) were collected as “TruMaps” applying corrections for background and peak overlaps. Prior to quantification, all elements in each spectrum were automatically identified. Trace elements (at% and wt% < 3σ) and system artifacts were excluded by automatic deconvolution from the spectrum. Among these system X-ray signals, the most intense observed were carbon and copper, which usually originated from the TEM grid, and trace iron signals from the TEM polepiece. Based on the large penetration depth of the electron beam into and through the nanoparticles in TEM, X-rays are originated up to the total thickness of the sample [20], obtaining EDS chemical elemental analysis from the bulk of the nanocrystals. Results are listed in wt% and at% with one and three standard deviations representing 68.3% and 99.7% confidence levels, respectively. Sample-average mass percent compositions of each element were calculated based on an intraclass correlation model part of the Analysis of Variance (ANOVA) methodology (see Supplementary Materials) [21].

### 2.4. Dynamic Light Scattering (DLS)

The hydrodynamic diameter and polydispersity index (PDI) of the ZnO-based NPS were determined by DLS using a Zetasizer Nano ZSP (Malvern Instruments, Worcestershire, UK) with λ = 633 nm at a measurement angle of 173° (backscatter). Data analysis was realized using Zetasizer software. Eethylene glycol was used as a solvent (refractive index 1.429 and viscosity of 16.1 mPa∙s at 25 °C) to convert the measured intensity/size distributions to number/size and volume/size distributions. Measurements were performed in disposable cuvettes (DTS 0012), using dispersed solutions of all samples in ethylene glycol (0.05 mg/mL) at 25 °C, previously sonicated (5 min) and filtrated (PTFE syringe filter, 0.45 µm). For each measurement, six runs were performed.

### 2.5. X-ray Photoelectron Spectroscopy (XPS)

X-ray photoelectron spectroscopy (XPS) analysis was realized to study the surface chemical composition in all samples, up to 10 nm beneath the surface, the maximum vertical depth resolution in XPS [22]. Measurements were performed using a Thermo Scientific Theta Probe Angle-Resolved XPS (ARXPS) system (ThermoFischer, East Grinstead, UK) equipped with monochromatic Al Kα X-rays source (1486.6 eV). XPS spectra were recorded with the hemispherical electron energy analyzer in fixed transmission mode, pass energy of 200 eV, and an energy channel step width of 1 eV and 0.05 eV, for general survey and high-resolution spectra, respectively. A dual flood gun was used for dual-charge neutralization in all analysis. Data acquisition and processing were performed using Thermo Scientific^TM^ Avantage Data System (software v.5.962, ThermoFischer, East Grinstead, UK). Additional charge compensation and chemical state identification of ZnO were performed for all samples using the modified Auger parameter (MAP) method [23], by fixing the Zn 2p_3/2_ line at a binding energy (BE) of 1021.0 eV for ZnO and comparing the modified Auger parameter (α′) value of ZnO to 2010.4 eV, (see Table S2) [24,25].

### 2.6. X-ray Powder Diffraction (XRPD)

X-ray diffraction in powder (XRPD) was performed on a PANalytical X’Pert Pro MRD XL diffractometer (Malvern Panalytical Ltd., Worcestershire, UK) equipped with a PIXcel 1D detector, W/Si graded parabolic mirror (for Cu Kα) PreFIX module, and Cu Kα radiation (λ = 1.54187 Å), operating at an accelerating voltage and emission current of 40 kV and 40 mA, respectively. XRD data were collected using a θ–2θ Bragg-Brentano geometry, a fixed divergence slit of 0.5°, and an offset of 0.004° for 2θ. Coupled 2θ-ω scans were acquired over the 5–105° 2θ range, with a 0.002° step size, and a time per step of 0.880 s (total measurement time of 12 h 26 min per sample). The instrumental contribution to the profile was obtained from a Si single crystal (001) standard, measured in the same conditions as all samples. The data collected were subsequently analyzed using the software FullProf [26] performing Rietveld refinements for all samples. The structural refinements were carried out in the space group *P*6_3_*mc* starting from atomic positions of pure ZnO [27]. The pseudo-Voigt profile function of Thompson, Cox, and Hastings [28] was used with the Finger’s treatment of the axial divergence [29]. The instrumental resolution function of the diffractometer was determined with a Si single crystal (001) standard, measured in the same conditions for all samples, and supplied in an IRF file (Instrument Resolution File) using the Gaussian and Lorentzian full-width at half-maximum (FWHM) components of the peak profile. Microstructural analysis based on the integral breadth method was used to obtain the average size and strain of diffracting domains. In all samples the following parameters were refined: scale factor, zero-point, cell parameters, isotropic temperature factors, atomic occupancies, oxygen atomic coordinates, and profile. The background was refined by adjusting the height of a set of pre-selected points for linear interpolation. Preferred orientation was observed due to the presence of aligned platelets and nanorods by the enhanced intensity of certain lattices planes *hkl* Bragg reflections, with the strongest effects observed for {*h*00}, {*hk*0}, and {00*l*} reflections. Intensity correction of these enhanced Bragg reflections was performed using the March–Dollase multiaxial function [30] included in Fullprof.

### 2.7. Photoluminescence Spectroscopy

Emission and excitation spectra were recorded with a Fluorolog-3 spectrometer (Horiba Jobin Yvon, Munich, Germany) equipped with a 450 W xenon lamp. For low temperature measurements, samples were placed in a quartz cell immersed in a liquid-nitrogen-filled Dewar flask previously purged with argon gas. Emission spectra using an excitation wavelength of 325 nm were performed with a long-pass filter KV580 to avoid second order Rayleigh scattering and emission. Site-selective spectroscopy of europium was performed with excitation and emission slits of the monochromator set to 1 nm bandpass to prevent the excitations of different Eu^3+^ sites. In addition, the relative quantum yields (QYs) were measured for all samples using a solution of Rhodamine 6G in ethanol (QY = 95%) as a reference material according to literature [31] (see Supplementary Materials Section S4).

### 2.8. Raman Scattering Spectroscopy

Raman scattering measurements were performed in the backscattering geometry using a Jobin Yvon/HORIBA LabRam ARAMIS Raman Spectrometer (Horiba Jobin Yvon, Edison, NJ, USA), equipped with a confocal microscope and a Peltier-cooled charge coupled device (CCD) detector (DU420A, ANDOR). Measurements were performed using an internal HeNe (633 nm) laser, an external 785 nm diode laser (Melles Griot, Carlsbad, CA, USA), and a 532 nm diode pump solid-state (DPSS) laser (mpc6000, Laser Quantum, Stockport, UK). All spectra were collected at room temperature in air with 15 s exposure time and ten accumulations.

## 3. Results and Discussion

### 3.1. Structural and Morphological Characterization by TEM and DLS

**Figure 1:**
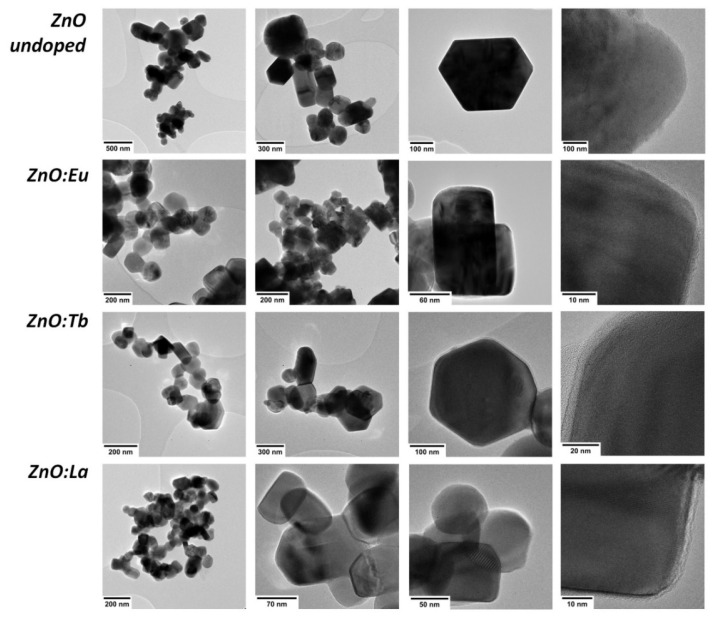
TEM images for different assemblies of nanocrystals from undoped ZnO NPs and lanthanide doped ZnO:Eu, ZnO:Tb, and ZnO:La NPs.

**Figure 2:**
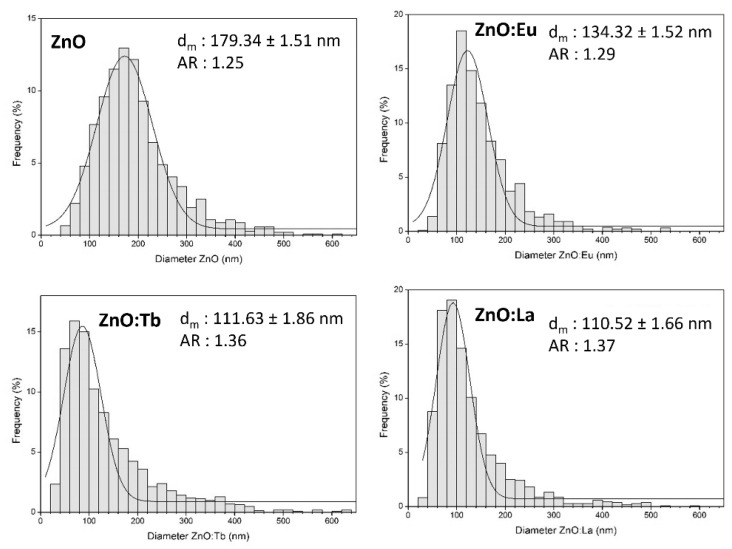
Size distribution histogram determined from TEM for different assemblies of nanocrystals from samples ZnO, ZnO:Eu, ZnO:Tb, and ZnO:La.

TEM micrographs from all samples exhibited in Figure 1 revealed highly crystalline particles of different morphologies, consisting of spherical, hexagonal, and rod-like shapes. In general, all size distributions are polydisperse and monomodal, ranging from 20 to 600 nm with a positive skewness (long tail on the right side). In particular, mean particle size revealed a decrease with doping from 180 nm for ZnO to 134 nm (ZnO:Eu), 110 nm (ZnO:Tb), and 111 nm (ZnO:La). Moreover, the aspect ratio factor increases from 1.25 for ZnO to 1.29 (ZnO:Eu), 1.36 (ZnO:Tb), and 1.37 (ZnO:La) (see Figure 2).

Apparently, doping might be influencing the particle growth, observed by the decrease in average size of 26% for Eu, and 38% for Tb and La. Morphological and size distribution features are collected in Table 1. In addition, the aspect ratio factor might be affected as well, since with doping it increases systematically at 3.2% for Eu, 8.8% for Tb, and 9.6% for La (Figure S1), revealing a change in shape from spherical to rod-like nanocrystals, associated with an increase in growth direction along the *c*-axis [32]. Size distributions determined by DLS (Figure S2) are monomodal with a Z-average size of 103 ± 26 nm, 217 ± 39 nm, 254 ± 41 nm, and 231 ± 58 nm, and a polydispersity index (PdI) of 0.84, 0.25, 0.75, and 0.48 for ZnO, ZnO:Eu, ZnO:Tb and ZnO:La NPs, respectively.

### 3.2. STEM-EDS Analysis

**Figure 3:**
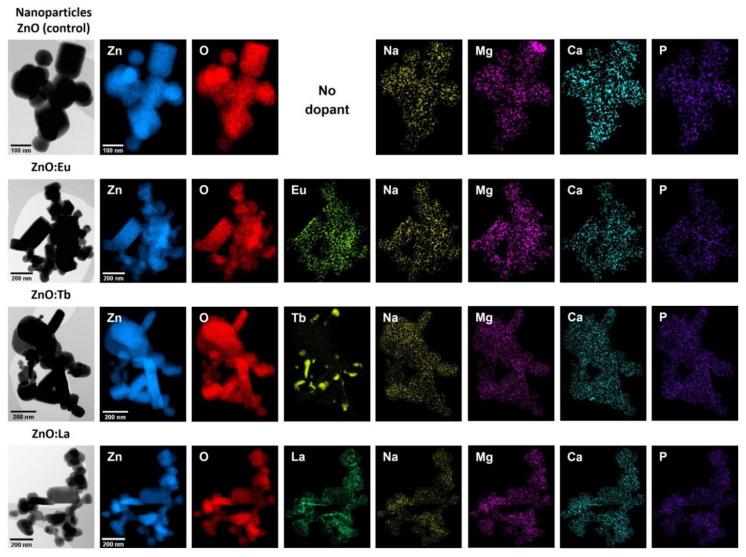
STEM images along with EDS elemental maps of undoped ZnO nanoparticles (control) and ZnO NPs doped with lanthanides (Eu, Tb and La).

STEM-EDS elemental mappings indicate a homogeneous distribution of Zn and O along with the intended lanthanide dopants in all samples analyzed. Additionally, small concentrations of non-intentional dopants were detected, including sodium (<1.36 at%), magnesium (<0.25 at%), calcium (<0.19 at%), phosphorous (<0.16 at%), potassium (<0.11 at%), and chlorine (<0.09 at%) (see Figure 3 and Figure S3 and Table S1). The presence of calcium and phosphorous can be explained due to the addition of whey as a reactant in the synthesis [15], which contains important concentrations of these elements in the form of calcium phosphate along with other constituents. Average elemental compositions (Table S1) were calculated from EDS elemental mappings and EDS maps sum spectra presented in Supplementary Materials Figures S4 and S5.

### 3.3. X-ray Photoelectron Spectroscopy (XPS)

**Figure 4:**
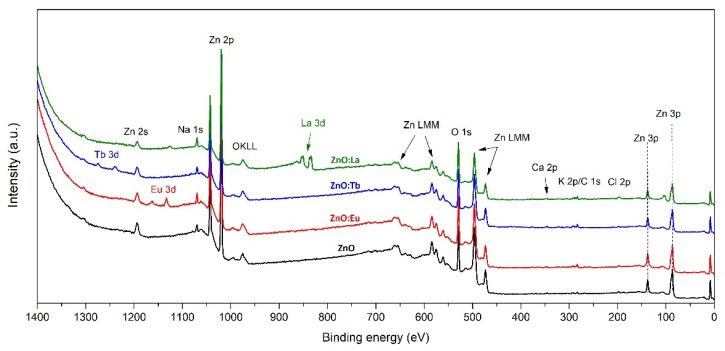
XPS spectra of Eu-, Tb-, and La-doped ZnO; and undoped ZnO.

To further investigate the chemical composition, XPS analysis was performed in all samples (ZnO, ZnO:Eu, ZnO:Tb, and ZnO:La). The wide energy spectra (0–1200 eV), presented in Figure 4, revealed the presence of Zn, O, Na, K, Ca and Cl, along with the intended lanthanide dopants (Eu, Tb, and La). In order to evaluate the elemental composition of the samples and the chemical states of the elements detected, high-resolution XPS spectra of Zn 2p, O 1s, Eu 3d, Tb 3d, La 3d, Na 1s, Ca 2p, K 2p, and Cl 2p photoemission lines were collected. Quantitative analysis of XPS spectra is presented in Table 2, where the at% of the intentional dopants is 1.81, 1.51, and 4.63% of Eu, Tb, and La, respectively, as expected from the synthesis. Binding energies of the main photoemission lines are listed in Table S2.

**Figure 5:**
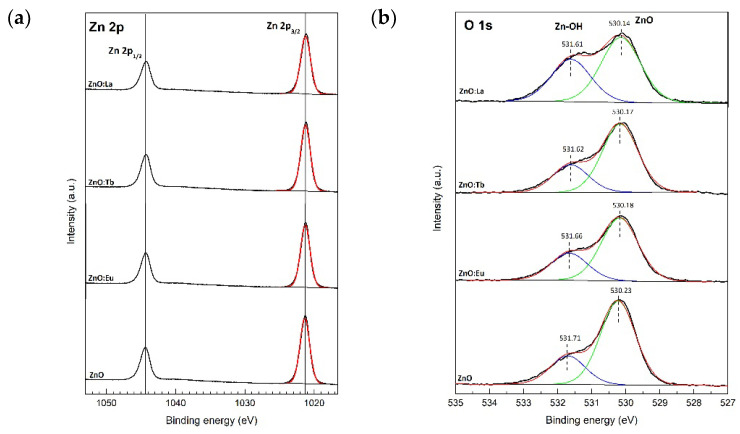
HRXPS spectra of (**a**) Zn 2p and of (**b**) O 1s core-level photoelectron line.

High resolution XPS spectra of Zn 2p and O 1s are depicted in Figure 5. The double peaks at 1021.3 and 1044.3 correspond to Zn 2p_3/2_ and 2p_1/2_ core-level photoelectron lines, respectively. The HRXPS spectra of O 1s exhibit a dominant emission at 530.2 eV, due to bulk oxygen in ZnO wurtzite structure, accompanied by a high energy shoulder at 531.6 eV, ascribed to oxygen from hydroxyl groups (Zn-OH) on Zn-polar (0001) or O-polar (000$\bar{1}$) faces [33].

The deconvolution and quantifications of O 1s from Zn-OH groups yield values of 25.4% (ZnO), 30.2% (ZnO:Eu), 28.9% (ZnO:Tb), and 39.5% (ZnO:La), consistent with typical values of hydroxyl groups on Zn (25.6%) or O (33.8%) polar faces. The zinc-to-oxygen atomic ratios (Zn/O) are 0.94, 0.77, 0.82, and 0.60 for ZnO, ZnO:Eu, ZnO:Tb, and ZnO:La, respectively. Including the non-intentional dopant sodium and the intentional lanthanide dopant, the Zn/O ratio is 1.02 (ZnO), 0.92 (ZnO:Eu), 0.95 (ZnO:Tb), and 0.79 (ZnO:La). A ratio below 1 might indicate a zinc deficiency due to zinc vacancies, or oxygen in excess due to oxygen interstitials in the ZnO crystal structure.

**Figure 6:**
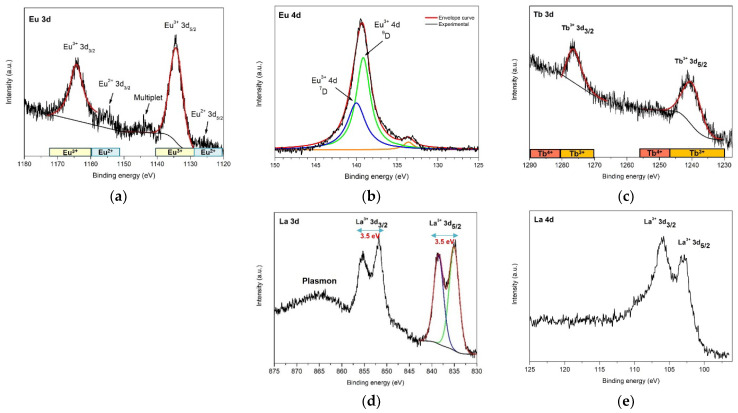
(**a**) HRXPS spectra of Eu 3d core-level photoelectron line from sample ZnO:Eu; (**b**) Eu 4d core-level photoelectron line from sample ZnO:Eu; (**c**) Tb 3d core-level photoelectron line from sample ZnO:Tb; (**d**) La 3d and (**e**) La 4d core-level photoelectron lines from sample ZnO:La.

The HRXPS spectrum of Eu 3d core level (Figure 6a) exhibits a doublet centered at 1134.3 eV and 1164.4 eV, corresponding to Eu^3+^ 3d_5/2_ and Eu^3+^ 3d_3/2_ photoelectron lines, respectively. The 30.1 eV separation between the doublets is due to the large spin-orbit coupling of Eu 3*d*^9^ 4*f*^6^ final state configurations. The binding energies of each peak and the large spin-orbit splitting of 30.1 eV, are well in agreement with trivalent Eu previously observed in Eu-doped ZnO and with Eu bonded to oxygen in Eu_2_O_3_ [34,35,36]. Interestingly, there are two additional low-intensity peaks that appear at 10 eV lower binding energy below Eu^3+^ 3d_5/2_ and 3d_3/2_ main groups, which can be assigned to divalent Eu 3d^9^ 4f^7^ final state configurations, Eu^2+^ 3d_5/2_ and 3d_3/2_ photoelectron lines. Finally, a less intense feature at 1142.7 eV corresponds to the part of the multiplet structure of Eu^3+^ 3d^9^ 4f^6^ final state [34].

To further support the previous assignments, the high-resolution spectrum of Eu 4d core-level was recorded. In general, the binding energies of Eu 4d spin-orbit doublets lie between 135 and 142.5 eV for Eu(III) and between 125 and 134 eV for Eu(II) [37,38,39]. The XPS spectra of Eu 4d core-level exhibit a more complex multiplet structure than spectra from Eu 3d core-level, due to the strong 4d–4f electrostatic interaction between the hole configuration 4d^9^ (with two possible states ^2^D_3/2_ and ^2^D_5/2_) and the open 4f^6^ sub-shell in the final state. Therefore, the photoionization of the Eu 4d core-level results in a *J* = *L* − *S* multiplet splitting, giving rise to the final state multiplets ^7^D and ^9^D [38,40].

In Figure 6b, the HRXPS spectra of Eu 4d core-level from sample ZnO:Eu exhibit a not-resolved peak centered at 139.5 eV, which can be deconvoluted into two peaks and assigned to the ^7^D and ^9^D multiplets. The satellite at 133.1 eV can be attributed to a shake-down peak of the trivalent Eu 3d^9^ 4f^6^ final state, or due to divalent Eu 3d^9^ 4f^7^ final state configurations.

Terbium 3d core-level spectrum (Figure 6c) exhibits the two final states Tb^3+^ 3d_5/2_ and Tb^3+^ 3d_3/2_, centered at 1239.97 eV and 1276.70 eV binding energies, respectively. The binding energies of each peak agree with trivalent terbium previously observed in Tb-doped ZnO and Tb bonded to oxygen in Tb_2_O_3_ [41,42,43]. A priori, no tetravalent terbium 3d photoelectron lines are observed in the spectrum [41].

Similarly to Eu and Tb, lanthanum 3d core-level spectrum (Figure 6d) exhibits well defined La 3d_5/2_ and La 3d_3/2_ spin-orbit doublets, situated at 835.06 and 851.8 eV, respectively, with a spin-orbit splitting of 16.7 eV. Both spin-orbit doublets show a satellite at 3.5 eV higher binding energy due to multiplet splitting from the strong mixing of the core hole final-state configuration 3d^9^4f^0^ with the charge transfer final state from the valence band (O 2p) to the La 4f orbital [44].

Interestingly, the intensity and energy separation of the La 3d satellites relative to the main peak are highly sensitive to the atomic coordination environment of La, and it is well documented that a different splitting of 4.2–4.6 eV is observed for La_2_O_3_ and 3.5–3.7 eV for La(OH)_3_ [45]. In the analyzed ZnO:La sample, the splitting of La 3d satellites is 3.50 eV, indicating the presence of mainly hydroxyl groups bonding to La atoms at the surface. Regarding the XPS La 4d spectrum (Figure 6e), similarly to Eu 4d, the exchange interaction between 4d and 4f states is stronger than between 3d and 4f states [46], resulting in a reduced spin-orbit splitting of the 4d state. Figure 6d,e shows La 4d core-level spectrum with the spin-orbit doublets La^3+^ 4d_5/2_ and 4d_3/2_ located at 103.10 and 106.00 eV, respectively. Finally, based on these observations, we can confirm the existence of La as a dopant in the ZnO wurtzite structure. Due to the presence of surface contaminant species such as a hydroxyl group, a reduction in the splitting of the La 3d spin-orbit doublets is observed in the experimental XPS spectra. High resolution XPS spectra from additional non-intentional dopants, Na, K, Ca, and Cl, were also recorded (see Figure S6).

Moreover, to understand the location and chemical environment of all elements present, depth profiling using angle resolved XPS (ARXPS) was performed on sample ZnO:Eu. (see Figure S7). In ARXPS, the surface sensitivity increases by increasing the angle between the sample and the analyzer, enabling the detection of electrons emitted at a shallower depth (near the surface). Depth profiling depicted in Figure S7 exhibits different trends in the atomic concentration of each element from bulk to surface. In particular, the concentration of Zn decreases systematically towards the surface, whereas oxygen from the lattice remains nearly constant.

On the contrary, oxygen from hydroxyl groups and C from adventitious carbon increase towards shallower depths, indicating that Zn-OH and adsorbed carbonaceous species have higher concentration on the surface of the particles, which confirms the previous assignment of O 1s core-level photoemission line centered at 531.2 eV to hydroxyl groups on polar surfaces of ZnO.

Regarding the dopants, Eu and Ca show a similar trend to Zn, with decreasing concentrations towards shallower depths. Moreover, Na remains constant, while Cl and K show higher atomic concentrations towards the surface.

The systematic high concentration of dopants observed near the surface could be indicative of atomic segregation, a common result when doping ZnO [47], while a lower concentration of certain elements near the surface is usually observed in non-stoichiometric ZnO crystals due to the presence of defects [48,49,50,51].

### 3.4. X-ray Powder Diffraction (XRPD)

**Figure 7:**
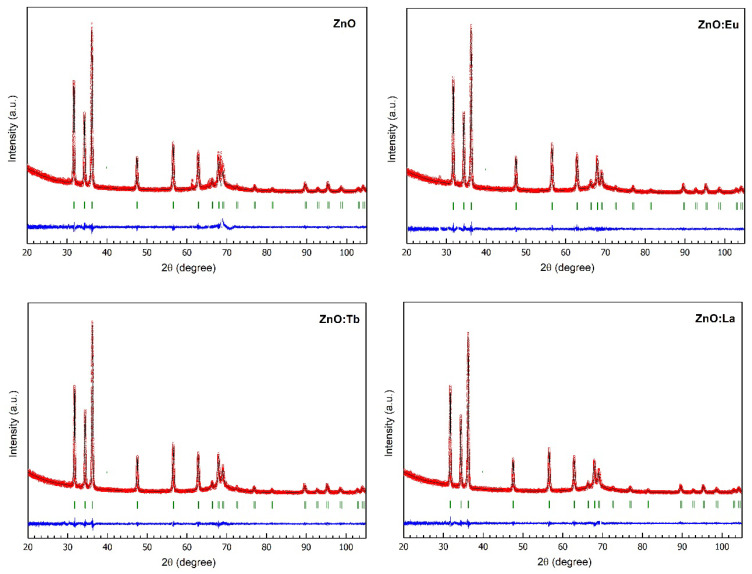
Rietveld refinement of ZnO, ZnO:Eu, ZnO:Tb, and ZnO:La samples. Experimental X-ray powder diffraction pattern is indicated by red dots, Rietveld-calculated profile by the black solid line and the difference between the observed and calculated patterns by the blue curve. Green vertical markers represent the calculated Bragg reflections of ZnO.

Bragg intensities were indexed in the hexagonal space group *P*6_3_*mc* by the Le Bail fit method [52], corresponding to wurtzite ZnO structure. No additional peaks due to secondary phases were observed, e.g., Ln_2_O_3_ (Ln: Eu, Tb or La). In final Rietveld refinements, fractional occupancies of oxygen and zinc were allowed to refine with the assumption that Zn is partially substituted by Na and lanthanide (Eu, Tb, La) atoms. The Rietveld refinement of the samples are displayed in Figure 7.

Cell structural refined parameters are summarized in Table 3. Selected bond distances and angles, domain size and average maximum strain are listed in Tables S3 and S4. From Table 3, it is observed that in lanthanide-doped ZnO samples, cell volume and *a*-cell parameters increase compared to the undoped ZnO sample. Regarding the *c*-parameter, a decrease is observed when Tb and La are present in ZnO, while it increases for ZnO:Eu. Due to the larger atomic radii of the dopants, Eu^3+^ (1.09 Å), Tb^3+^ (1.06 Å), La^3+^ (1.17 Å), and Na^+^ (1.13 Å), compared to Zn^2+^ (0.74 Å), an expansion of the hexagonal wurtzite unit cell would be expected along *c* and *a*-axes. The contrary behavior observed in the reduction of the *c*-cell parameter in Tb- and La-doped ZnO samples has been previously reported for ZnO doped with neodymium, gadolinium, and erbium; and explained based on the formation of defects such as Zn or O vacancies in order to maintain the electrical neutrality of the crystal structure [47,48,49,50]. The mechanism is the following: three Zn^2+^ cations are substituted by two Ln^3+^ cations and the formation of a neutral zinc vacancy (*V*_Zn_) [49].

3 Zn^2+^ → 2 Ln^3+^ + *V*_Zn_

The resulting Zn fractional occupancies exhibit values below 1 in all samples, which is a good indication towards the formation of Zn vacancies. Average domain sizes and microstrain were obtained using the integral breadth method and the input file containing the IRF file in Fullprof. The average domain sizes show a minimum value of 40 nm for undoped ZnO and a maximum value of 43 nm for the ZnO:Eu sample. Microstrain in undoped ZnO shows a value of 13.96%, while in Eu-doped ZnO an increase of 0.5% is observed. Differently, in Tb- and La-doped ZnO samples, microstrain values decrease by 2.6 and 2.5%, respectively.

### 3.5. Photoluminescence Spectroscopy

**Figure 8:**
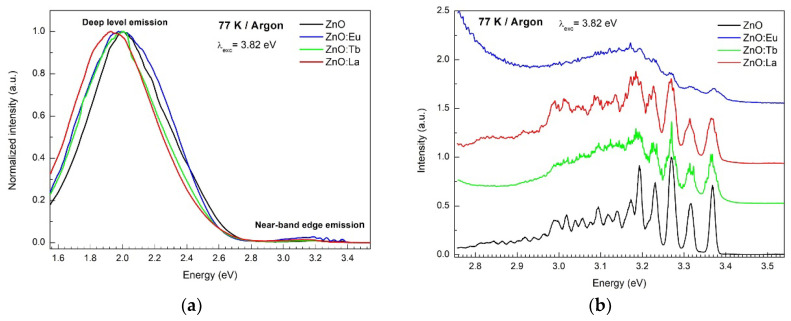
(**a**) PL emission spectra performed at 77 K of ZnO, ZnO:Eu, ZnO:Tb, and ZnO:La NPs; (**b**) PL emission spectra magnified in the near-band edge region.

PL emission spectra depicted in Figure 8 were recorded using an excitation energy of 3.82 eV (325 nm). The spectra performed at 77 K consist in general of a low-intensity UV band centered at 3.19 eV and an intense and broad emission in the visible region of the spectrum, centered around 2.00 eV. The UV-band corresponds to the near-band edge (NBE) emission from the recombination of free and bound excitons in ZnO [53]. The broad visible band is ascribed to the deep level emission from additional deep states in the bandgap, associated with the recombination of carriers at intrinsic defects in the crystal structure and at adsorbed species on the ZnO surface [53]. Based on recent investigations, this broad band is the result of the combination of a yellow emission centered at 2.00 eV and a green emission at 2.41 eV, ascribed to reduced species of oxygen ($O_{2}^{2-}$) on the ZnO surface and to single-charged oxygen vacancies $V_{o}^{+}$ in the crystal structure of ZnO [54,55]. The PL spectra obtained at 298 K (Figure S8) show band broadening and maxima shifts for all samples compared to the spectra obtained at 77 K. In particular, the PL spectra of Eu doped ZnO show a sharp peak at 2.03 eV (611.8 nm), corresponding to the ^5^D_0_ → ^7^F_2_ transition from Eu^3+^ ions [56].

The luminescence mechanism of lanthanides in doped nanocrystals depends on their location in the host lattice. Weak perturbations of the local structure can result in significant changes in the local crystal-field strength and site symmetry of the lanthanide ions [56]. Based on different crystal field surroundings, the multiple non-equivalent sites of Eu^3+^ ions created are characterized by exhibiting emission and excitation spectra with different intensities and crystal field splitting of the bands (Stark sublevels) [57]. In the hexagonal wurtzite structure of ZnO, Zn**^2+^** ions are located at a lattice site with *C*_3*v*_ symmetry, tetrahedrally coordinated to oxygen ions. If Zn^2+^ ions (0.74 Å) are substituted by Eu^3+^ ions with a larger radius (1.09 Å), a significant lattice distortion is expected to occur due to induced strain and defects, created to maintain the overall electric charge neutrality [58]. The photoluminescence of europium(III) ions is characterized by intense bands due to ^5^D_0_ → ^7^F_J_ transitions (*J* = 0–6) from the ^5^D_0_ excited state to the *J* levels of the ^7^F ground term. The different number of transitions between two ^2*S*+1^*L_J_* terms depend on the point group symmetry of the Eu^3+^ ion. If a lowering in symmetry takes place, the number of allowed transitions increases due to the relaxation of the selection rules.

**Figure 9:**
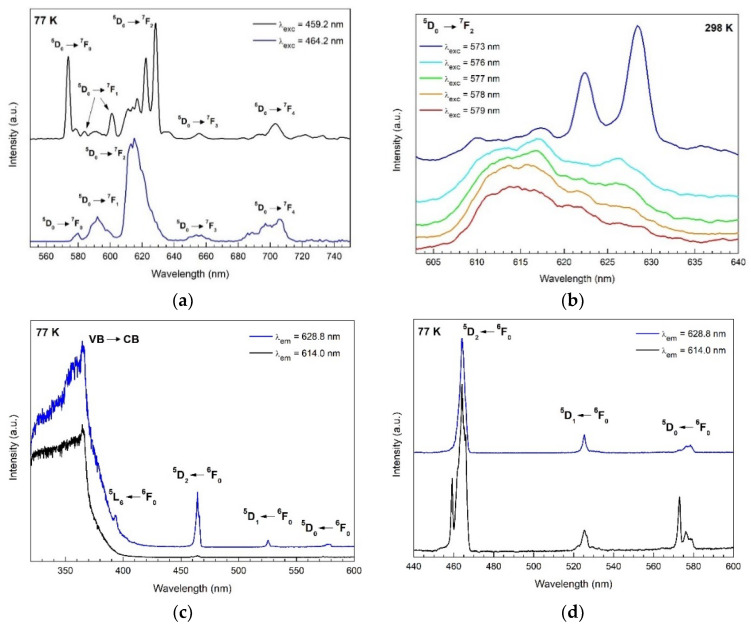
(**a**) Site-selective emission spectra of Eu^3+^ ions in ZnO:Eu at 77 K; (**b**) Site-selective emission spectra of ^5^D_0_ → ^7^F_2_ transitions from Eu^3+^ ions in ZnO:Eu at 298 K; (**c**) Site-selective excitation spectra of Eu^3+^ ions in ZnO:Eu at 77 K; (**d**) Magnified spectra (**c**) in the 440–600 nm region.

In this work, resolution site-selective emission and excitation spectroscopy were performed at 77 and 298 K. Analysis of the number of 4f-4f transitions observed in the spectra was used for the determination of the symmetry point group of different Eu^3+^ ion sites. Site-selective emission spectroscopy was realized by exciting the ^5^D_2_ ← ^7^F_0_ transition of two different Eu^3+^ symmetry sites under 464 and 459 nm excitation wavelengths. Under excitation at 459.2 nm, sharp emission lines are observed attributed to intra-4f transitions ^5^D_0_ → ^7^F_J_ (*J* = 0–6) of Eu^3+^ ions, as labeled in Figure 9a (and Figure S9a at 298 K). Based on the number of transitions observed in the spectra, the point group symmetry of the Eu^3+^ ion was deduced as follows: for transition ^5^D_0_ → ^7^F_0_ one band is observed at 573.8 nm. Due to the non-degeneracy of the ^7^F_0_ and ^5^D_0_ levels, a maximum of one peak is expected for each symmetry site of Eu^3+^ ions [56].

The transition ^5^D_0_ → ^7^F_1_ shows two bands at 583.8 and 601.0 nm. Related to the electric dipole transition ^5^D_0_ → ^7^F_2_, three lines with the highest intensity are observed in the spectrum at 616.8, 622.4, and 628.2 nm. Notice that the left shoulder at 611.2 nm is due to the ^5^D_0_ → ^7^F_2_ transition from a different site of lower symmetry (see blue curve below Figure 9a).

In general, the majority of ^5^D_0_ → ^7^F_J_ (*J* = 0–6) transitions observed are of an electric dipole nature, except for the magnetic dipole ^5^D_0_ → ^7^F_1_ transition, for which intensity is independent to the site symmetry. According to the Judd–Ofelt theory, electric dipole transitions are only allowed for a Eu^3+^ ion at a site without a center of symmetry (inversion center). Therefore, from the 32 crystallographic point groups, the 21 meeting this criterion are: *C*_6_, *C*_6*v*_, *D*_6_, *D*_3*h*_, *D*_3_, *C*_3*v*_, *C*_3*h*_, *C*_3_, *D*_4_, *C*_4*v*_, *C*_4_, *S*_4_, *D*_2*d*_, *D*_2_, *C*_2*v*_, *C*_2_, *C*_s_, *C*_1_, *O*, *T_d_*, and *T* [59].

Additionally, according to the electric dipole selection rule, the ^5^D_0_ → ^7^F_0_ transition is only allowed for the following 10 site symmetries, *C*_s_, *C*_1_, *C_n_*, and *C_nv_* (*n* = 2, 3, 4, 6) [60,61]. Therefore, the only point groups without an inversion center and showing one band for the ^5^D_0_ → ^7^F_0_ transition, two bands for the ^5^D_0_ → ^7^F_1_ transition and three bands for the ^5^D_0_ → ^7^F_2_ transition are *C*_3_ and *C*_3*v*_. Since Zn^2+^ ions are located at a lattice site with *C*_3*v*_ symmetry in the ZnO crystal lattice, the results confirm the successful incorporation of europium by the substitution of Zn^2+^ ions.

In the spectra under excitation at 464.2 nm (blue curve in Figure 9a), broader emission bands are observed. The unresolved and inhomogeneous broadening is an indication of the existence of multiple non-equivalent sites of Eu^3+^ ions with lower symmetry. Looking at the space group *P*6_3_*mc* of hexagonal ZnO, an additional cation site for Eu^3+^ ions can be the 6*c* Wyckoff position with a *C*_s_ point group symmetry in Schoenflies notation (*m* point group symmetry in Hermann–Mauguin notation) [62]. Based on the theoretical branching rules of the 32-point groups from Butler [63], C_s_ is one of the two possible point groups with lower symmetry descending from *C*_3*v*_ (*C*_3*v*_ → *C*_s_ → *C*_1_).

Site-selective excitation spectra were recorded at 77 (Figure 9c) and 298 K (Figure S9) by monitoring the ^5^D_0_ → ^7^F_2_ transition from two different Eu^3+^ sites at 628.2 and 614.0 nm for 298 K, and at 628.8 and 614.0 nm for 77 K. The excitation spectra at 298 K (Figure S9b) show five excitation bands while monitoring the emission at 614.0 nm (maximum intensity of the ^5^D_0_ → ^7^F_2_ transition assigned to the low symmetry site). The transitions correspond to the excitation from the ground state ^7^F_0_ to the different excited states ^5^L_6_, ^5^D_3_, ^5^D_2_, ^5^D_1_, and ^5^D_0_ of the Eu^3+^ ions, centered at 393.8, 413.8, 464.2, 533.0, and 577.4 nm, respectively. In comparison, when monitoring the emission at 628.2 nm (the maximum intensity of the ^5^D_0_ → ^7^F_2_), an additional peak is observed for each of the ^5^D_2_ ← ^7^F_0_ and ^5^D_0_ ← ^7^F_0_ transitions_,_ centered at 459.0 and 572.8 nm, respectively. For the ^5^D_0_ ← ^7^F_0_ transition, two bands are observed, a narrow band at 572.8 nm and a broad band from 574.4 nm to 581.0 nm, corresponding to the high symmetry *C*_3*v*_ and lower symmetry *C*_s_ sites of Eu^3+^ ions, respectively. The broadness of the second band suggests that multiple non-equivalent sites of low symmetry *C*_s_ and similar energy are coexisting in the sample.

Additionally, to the excitation bands of Eu^3+^ ions, a broad high energy band is observed in both spectra (Figure S9b), centered at 373.0 nm (3.32 eV) and 374.2 nm (3.31 eV), by monitoring the emission at 628.2 and 614.0 nm, respectively. The origin of the band is due to the transition between the valence band and conduction band in ZnO, probably emitting through defects located at the emission wavelength. Previous research groups have also attributed this band to an energy transfer from ZnO to Eu^3+^ ions [64,65].

Site-selective excitation spectra at 77 K exhibit the same number of excitation bands, with clearer Stark splitting of the bands. In order to identify further the number of sites of Eu^3+^ ions, site-selective PL spectroscopy was performed by selectively exciting the ^5^D_0_ ← ^7^F_0_ transition for each site and analyzing the ^5^D_0_ → ^7^F_2_ transitions. As observed from Figure 9b, the emission from ^5^D_0_ → ^7^F_2_ transitions under the excitation of 573 nm exhibits three narrow bands characteristic of the higher symmetry site *C*_3*v*_ previously observed when exciting the ^5^D_2_ ← ^7^F_0_ transition at 459.0 nm in the PL spectra (Figure 9a,d). Differently, excitations between 576 and 579 nm show a broader emission band without resolved Stark splitting previously attributed to the low symmetry site *C*_s_ when exciting the ^5^D_2_ ← ^7^F_0_ transition at 464.0 nm in the PL emission spectra (Figure 9a,d).

Since at least five lines are expected from a low symmetry *C*_s_ point group, the Stark splitting of the bands is smaller than from a high symmetry *C*_3*v*_ point group exhibiting three lines. A smaller Stark splitting indicates that in the low symmetry site *C*_s_ a weaker crystal field is interacting with the Eu^3+^ ions [66]. Related to the possible existence of Eu^2+^ ions, the fluorescence spectra of divalent europium in ZnO are completely different to Eu^3+^ and would show a very broad emission at 475 nm due to d–f transitions (4f^6^ 5d → 4f^7^) [67].

**Figure 10:**
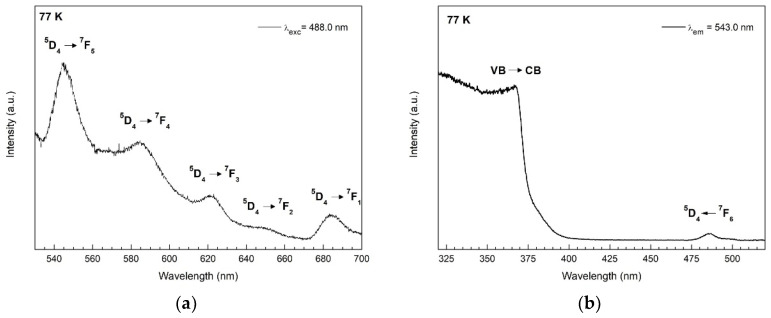
Emission (**a**) and excitation (**b**) spectrum of Tb^3+^ ions in ZnO:Tb at 77 K.

For the Tb doped ZnO sample, the excitation spectrum was performed monitoring the emission at 543 nm attributed to the ^5^D_4_ → ^7^F_5_ transition of Tb^3+^ ions. The only peak observed is associated with the transition from the ground state ^7^F_5_ to the ^5^D_4_ excited level at 488.0 nm (see Figure 10). The additional excitations from the ground state to higher excited levels that take place below 400 nm are not visible due to the overlap with the excitation of the valence band-to-conduction band transition. The emission spectrum was obtained using the excitation wavelength of 488.0 nm of the ^5^D_4_ ← ^7^F_5_ transition (Figure 10a). Here, five emission bands are observed at 544.0, 584.7, 622.1, 649.9, and 684.2 nm, corresponding to the transition from the excited level ^5^D_4_ to ^7^F_5_, ^7^F_4_, ^7^F_3_, ^7^F_2_, and ^7^F_1_, respectively [68,69].

### 3.6. Raman Scattering Spectroscopy

**Figure 11:**
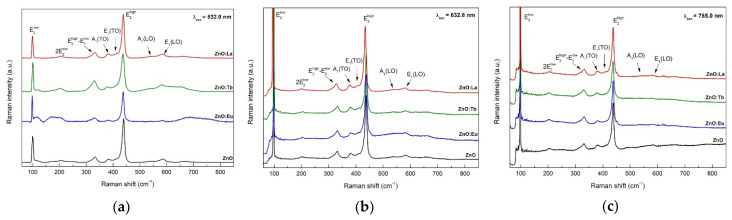
Raman spectra of ZnO, ZnO:Eu, ZnO:Tb, and ZnO:La under 532.0 nm (**a**); 632.8 nm (**b**); and 785.0 nm (**c**) excitation wavelength.

Raman study was carried out for the characterization and estimation of the crystallinity of the synthesized ZnO NPs (see Raman spectra in Figure 11). Wurtzite ZnO belongs to the space group $C_{6v}^{4}$ (in Schoenflies notation) with two formula units per primitive cell and atoms occupying *C*_3*v*_ symmetry sites. Group theory predicts six modes of zone-center optical phonons, $\Gamma_{\mathrm{opt}}=A_{1}+E_{1}+2E_{2}+2B_{1}$ [70,71]. *A*_1_ and *E*_1_ modes are polar modes with different frequencies for the transverse-optical (TO) and longitudinal-optical (LO) phonons. *E*_2_ are two nonpolar modes with different frequency $E_{2}^{\mathrm{high}}$ and $E_{2}^{\mathrm{low}}$, associated with the vibrations of the oxygen sublattice and zinc sublattice, respectively [72]. Raman spectra depicted in Figure 11 show several bands around 99, 384, 410, 439, 585, and 592 cm^−1^ ascribed to $E_{2}^{\mathrm{low}}$, *A*_1_(TO), *E*_1_(TO), $E_{2}^{\mathrm{high}}$, *A*_1_(LO), and *E*_1_(LO), first-order modes of hexagonal ZnO, respectively. Additionally, second-order scattering modes are observed at 205, 333, and 536 cm^−1^ assigned to $2E_{2}^{\mathrm{low}}$, $E_{2}^{\mathrm{high}}-E_{2}^{\mathrm{low}}$, and $2B_{1}^{\mathrm{low}}$ or 2 longitudinal acoustic (LA) overtones [72], respectively.

Comparing Raman spectra using different excitation wavelengths, 532 nm (2.33 eV) Figure 11a, 633 nm (1.96 eV) Figure 11b, and 785 nm (1.58 eV) Figure 11c, changes in relative intensities are observed, especially for $E_{2}^{\mathrm{low}}$ and $E_{2}^{\mathrm{high}}$ modes. According to the literature [57], experimental scattering efficiencies (or intensities) of first-order modes increase with increasing laser excitation energies, closer to resonant conditions with the energy gap of ZnO (3.3 eV). As from previous Raman spectra, we can observe an increase in the intensity of the $E_{2}^{\mathrm{high}}$ mode with larger excitation laser energy, whereas the intensity of $E_{2}^{\mathrm{low}}\;$ mode is constant. The reason behind this is because near the resonant condition (VB to CB transition), the intensity of $E_{2}^{\mathrm{high}}$ mode is highly influenced since the valence band is composed almost exclusively of oxygen 2*p* wavefunctions [73].

## 4. Conclusions

In summary, we have reported the effect of doping ZnO NPs with rare-earth elements (Eu, Tb, and La) by using whey as a chelating agent, a promising green, effective, and affordable chemistry synthesis of doped ZnO NPs. All the nanoparticles exhibited highly crystalline structure ranging from 20 to 600 nm, with different morphologies. The doping has shown influence in particle growth, observed by a decrease in average size of 26% for Eu and 38% for Tb and La, and a systematically increase of the aspect ratio factor from Eu to Tb and La. A homogeneous distribution of Zn and O, along with the intended lanthanide dopants, was detected by STEM-EDS analysis, along with small concentrations of non-intentional dopants (Na, Mg, Ca, P, K, and Cl), due to the addition of whey as a reactant in the synthesis. This agreed with the chemical composition revealed by XPS the presence of Zn, O, Na, K, Ca, and Cl, along with the intended lanthanide dopants, where the atomic concentration of the intentional dopants is 1.81, 1.51, and 4.63% of Eu, Tb, and La, respectively, as expected from the synthesis. Here, ARXPS enabled us also to locate a decreasing concentration of Zn towards the surface, whereas oxygen from the lattice remained nearly constant. Moreover, PL emission spectra from the samples were measured to study the effect on the luminescence mechanism of lanthanides in doped nanocrystals due to their location in the host lattice. We can conclude that Eu, Tb, and La-doped ZnO NPs are safer, more sustainable, and more environmentally compatible than Cd-based quantum dots, while the synthesis using whey as a chelating agent paths new roads for the obtention of eco-friendly and fluorescent semiconductor nanomaterials.

## Figures and Tables

**Table 1:** Morphological and size distribution from TEM micrographs of ZnO, ZnO:Eu, ZnO:Tb, and ZnO:La NPs.

Sample	ZnO	ZnO:Eu	ZnO:Tb	ZnO:La
**Morphologies**	Rod, hexagonal, and spherical shape			
**Particle size distribution (PSD)**				
Geometric mean size (nm)	179.34 ± 1.51	134.32 ± 1.52	111.63 ± 1.86	110.52 ± 1.66
Aspect ratio (AR)	1.25 ± 0.24	1.29 ± 0.26	1.36 ± 0.34	1.37 ± 0.36
AR change	0%	3.2%	8.8%	9.6%
Span (dispersion)	1.09	1.16	2.02	1.59
Number of Modes Mode center (nm)	1 mode 170	1 mode 110	1 mode 70	1 mode 90
Decreased size	0%	−26%	−38%	−38%

**Table 2:** Elemental composition of ZnO, ZnO:Eu, ZnO:Tb and ZnO:La samples.

	Elemental Composition (at%)							Atomic Ratio		
Sample	Zn 2p_3/2_	O 1s	Ln (Eu/Tb/La)	Na 1s	Ca 2p_3/2_	K 2p_3/2_	Cl 2p_3/2_	Zn/O	Zn(Na,Ln)/O	Ln/Zn
ZnO	45.04	48.13	-	3.84	0.75	0.78	1.46	0.94	1.02	-
ZnO:Eu	38.74	50.10	1.81	5.48	0.61	1.36	1.84	0.77	0.92	0.05
ZnO:Tb	40.49	49.29	1.51	4.67	1.06	1.68	1.29	0.82	0.95	0.04
ZnO:La	31.65	52.92	4.63	5.77	0.76	1.69	2.58	0.60	0.79	0.15

**Table 3:** Cell parameters, conventional Rietveld reliability factors, and structural parameters obtained from Rietveld refinements of ZnO, ZnO:Eu, ZnO:Tb, and ZnO:La.

	ZnO	ZnO:Eu	ZnO:Tb	ZnO:La
**Space Group**	*P*6_3_*mc*	*P*6_3_*mc*	*P*6_3_*mc*	*P*6_3_*mc*
Cell parameters				
a (Å)	3.24976 (2)	3.25003 (2)	3.25023 (2)	3.25019 (2)
c (Å)	5.20536 (3)	5.20544 (3)	5.20483 (3)	5.20487 (3)
V (Å^3^)	47.608 (0)	47.617 (0)	47.620 (0)	47.616 (0)
Density (g/cm^3^)	5.796	5.925	6.195	6.020
**Conventional Rietveld** **Reliability Factors (%)**				
*R*_p_	6.30	5.80	5.79	5.75
*R*_wp_	8.69	7.87	7.88	7.87
R_exp_	7.33	7.31	7.13	7.31
*R*_Bragg_	2.58	2.53	2.48	2.51
*R*_F_	2.22	2.60	2.30	2.48
*Χ* ^2^	1.40	1.16	1.22	1.16
**Structure**				
O atomic coordinate in *z*-axis	0.38710 (30)	0.38746 (28)	0.38294 (25)	0.38130 (24)
Zn occupancy	0.929 (3)	0.918 (2)	0.909 (2)	0.904 (2)
O occupancy	0.998 (9)	1.000 (3)	1.007 (2)	1.000 (2)
Na occupancy	0.021 (7)	0.021 (7)	0.035 (7)	0.028 (6)
Ln occupancy (Eu, Tb, La)	-	0.015 (1)	0.028 (1)	0.016 (1)
Zn B_iso_ (Å^2^)	0.355 (8)	0.739 (9)	0.819 (8)	1.085 (8)
O B_iso_ (Å^2^)	0.945 (38)	1.184 (41)	0.964 (35)	1.474 (37)
Na B_iso_ (Å^2^)	0.897 (8)	0.991 (9)	0.809 (8)	1.075 (8)
Ln B_iso_ (Å^2^) (Eu, Tb, La)	-	0.994 (9)	0.799 (8)	0.985 (8)

## Data Availability

Not applicable.

## Supplementary Materials

The following are available online at https://www.mdpi.com/article/10.3390/nano12132265/s1, Figure S1: Aspect ratio distribution of samples ZnO, ZnO:Eu, ZnO.Tb, and ZnO:La determined by TEM; Figure S2: Size distribution of samples ZnO, ZnO:Eu, ZnO.Tb, and ZnO:La determined by DLS; Figure S3: EDS elemental maps (multicolor Trumaps) of undoped ZnO nanoparticles (NPs) and doped ZnO NPs with lanthanides (Eu, Tb, and La); Figure S4: EDS map sum spectrum of ZnO (a) and ZnO:Eu (b). (c) Insets of (b) magnified in the 4.6–8.3 eV region, shows peak shapes of Eu L line series fitted to the spectrum by the Filtered Least Squares approach. EDS spectrum images (SIs) were acquired with 199 × 166-pixel dimensions and 62.4 ms dwell-time; Figure S5: EDS map sum spectrum of ZnO:Tb (a) and ZnO:La (b). (c) Inset of (a) magnified in the 5.0–9.5 eV region, shows peak shapes of Tb L line series fitted to the spectrum by the Filtered Least Squares approach. EDS SIs were acquired with 1024 × 1024-pixel dimensions and 35 μs dwell-time. (d) Insets of (b) magnified in the 4.0–6.2 eV region, shows peak shapes of La L line series fitted to the spectrum by the Filtered Least Squares approach. EDS SIs were acquired with 512-pixel × 512-pixel dimensions and 50 μs dwell-time; Figure S6: HRXPS spectra of (a) Na 1s; (b) K 2p3/2; (c) Ca 2p3/2 (c); and (d) Cl 2p3/2 core-level photoelectron lines from ZnO, and Eu, Tb, La doped ZnO samples; Figure S7: (a) ARXPS of Zn 2p, O 1s lattice, O 1s -OH and adventitious C 1s. (b) ARXPS (right) Na 1s, K 2p_3/2_, Cl 2p_3/2_, Ca 2p_3/2_, and Eu 3d_5/2_, from sample ZnO:Eu; Figure S8: PL emission spectra performed at 298 K of ZnO, ZnO:Eu, ZnO:Tb, and ZnO:La NPs; Figure S9: (a) Site-selective emission spectra of Eu^3+^ ions in ZnO:Eu at 298. (b) Site-selective excitation spectra of Eu^3+^ ions in ZnO:Eu at 298 K; Table S1: Average elemental composition of undoped and lanthanide-doped ZnO NPs listed in at% with one standard deviation (σ) (obtained from STEM-EDS measurements); Table S2: Binding energies of Zn 2p_3/2_, O 1s, O 1s (Zn-OH), C 1s (C-C/C-H), Na 1s, Ca 2p_3/2_, K 2p_3/2_, and Cl 2p_3/2_; Table S3: Selected bond distances and angles of ZnO, ZnO:Eu, ZnO:Tb, and ZnO:La; Table S4: Domain size and average maximum strain of ZnO, ZnO:Eu, ZnO:Tb, and ZnO:La samples.
