# Supplementary material for: Lanthanide (Eu, Tb, La)-Doped ZnO Nanoparticles Synthesized Using Whey as an Eco-Friendly Chelating Agent
Source: Nanomaterials (Basel). 2022 Jun 30;12(13):2265. doi: 10.3390/nano12132265 (PMC9268008; doi:10.3390/nano12132265)
Supplement: Supplementary file 1 [file nanomaterials-12-02265-s001.zip › nanomaterials-1747274-supplementary.pdf]

Supporting Information

Lanthanide (Eu, Tb, La)-Doped ZnO Nanoparticles Synthesized using Whey as an Eco-Friendly Chelating Agent

Carolina Picasso [1], Yolanda Salinas [2,*], Oliver Brüggemann [2], Markus Clark Scharber [3], Niyazi Serdar Sariciftci [3], Olavo D. F. Cardozo [4,5], Eriverton S. Rodrigues [6,7], Marcelo S. Silva [7], Andreas Stingl [5] and Patricia M. A. Farias [6,*]

[1] Institute of Inorganic Chemistry, Johannes Kepler University Linz, Altenberger Straße 69, 4040 Linz, Austria; carolina.picasso@jku.at (C.P.)
[2] Institute of Polymer Chemistry (ICP), Johannes Kepler University Linz, Altenberger Straße 69,
4040 Linz, Austria; oliver.brueggemann@jku.at (O.B.)
[3] Linz Institute for Organic Solar Cells (LIOS), Physical Chemistry Institute, Johannes Kepler University Linz, Altenberger Straße 69, 4040 Linz, Austria; markus_clark.scharber@jku.at (M.C.S.); serdar.sariciftci@jku.at (N.S.S.)
[4] Post-Graduate Program on Electrical Engineering, Federal University of Pernambuco, Cidade Universitaria, Recife 50670-901, Brazil; olavo.cardozo@ufpe.br (O.D.F.C.)
[5] Phornano Holding GmbH, Kleinengersdorferstrasse 24, 2100 Korneuburg, Austria; andreas.stingl@phornano.com (A.S.)
[6] Post-Graduate Program on Material Sciences, Federal University of Pernambuco, Cidade Universitaria, Recife 50670-901, Brazil; eriverton.rodrigues@if-sertao-pe.edu.br (E.S.R.)
[7] Federal Institute of Education, Science and Technology of Sertão Pernambucano, Salgueiro 56000-000, Brazil; marcelo.silva@ifsertao-pe.edu.br (M.S.S.)
* Correspondence: yolanda.salinas@jku.at (Y.S.); patricia.mafarias@ufpe.br (P.M.A.F.)

1. Structural and morphological analysis from TEM-EDS analysis and DLS

1.1. Size distribution by TEM and DLS

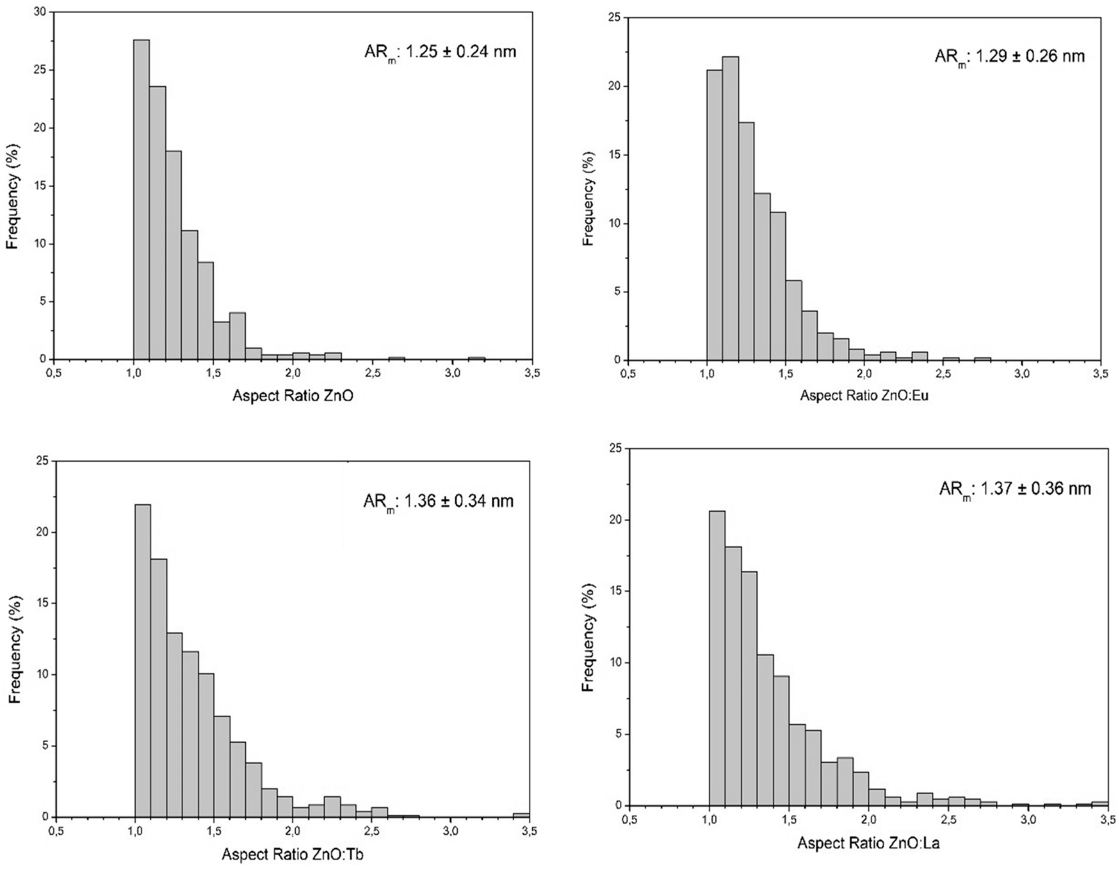

Figure S1. Aspect ratio distribution of samples ZnO, ZnO:Eu, ZnO:Tb, and ZnO:La determined by TEM.

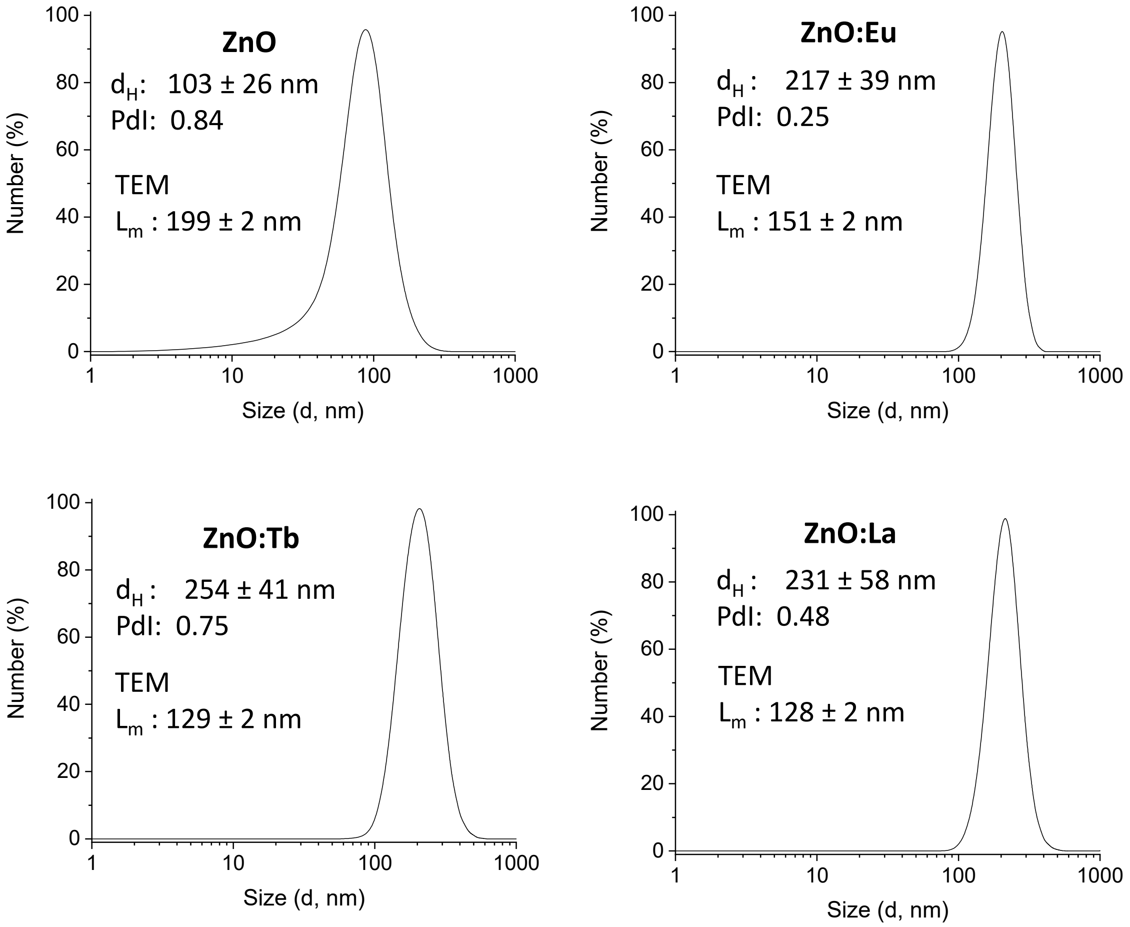

Figure S2. Size distribution of samples ZnO, ZnO:Eu, ZnO:Tb, and ZnO:La determined by DLS.

1.2. STEM-EDS analysis

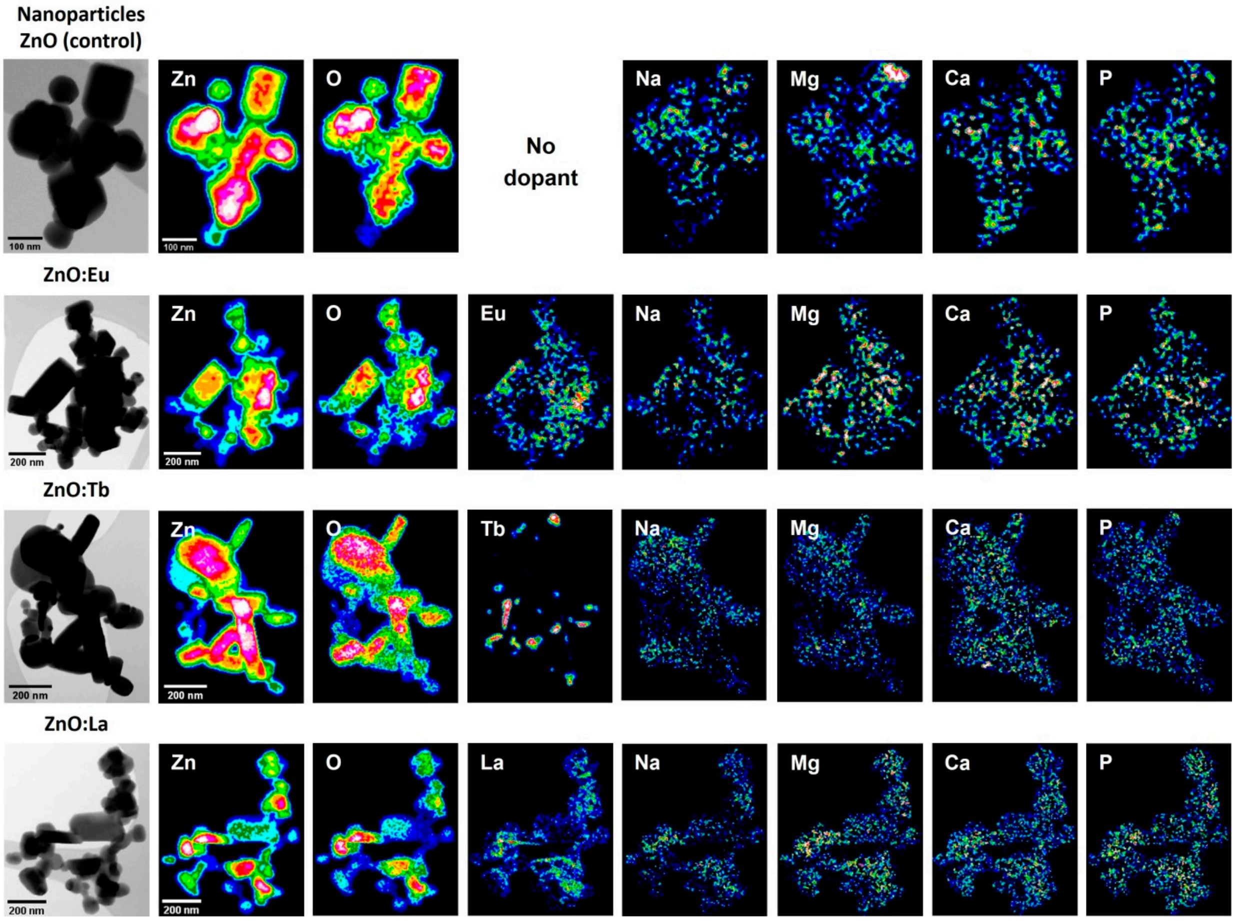

Figure S3. EDS elemental maps (multicolor Trumaps) of undoped ZnO nanoparticles (NPs) and ZnO NPs doped with lanthanides (Eu, Tb, and La).

Table S1. Average elemental composition of undoped and lanthanide-doped ZnO NPs listed in at% with

NPs	Zn (at% ± σ)	O (at% ± σ)	Ln[1] (at% ± σ)	Na (at% ± σ)	Ca (at% ± σ)	Mg (at% ± σ)	P (at% ± σ)	K (at% ± σ)	Cl (at% ± σ)
ZnO	45.70±0.06	52.82±0.18	-	0.92±0.13	0.16±0.01	0.25±0.02	0.16±0.02	-	-
ZnO:Eu	46.62±0.16	51.74±0.37	0.34±0.04	0.76±0.40	0.19±0.04	0.22±0.06	0.14±0.05	-	-
ZnO:Tb	60.01±0.04	37.35±0.12	1.42±0.01	0.77±0.10	0.12±0.01	<0.1	0.16±0.01	0.07±0.01	0.09±0.01
ZnO:La	53.81±0.04	43.70±0.12	0.47±0.01	1.36±0.11	0.18±0.01	0.19±0.02	0.15±0.01	0.11±0.01	0.04±0.01

standard deviation (σ) obtained from STEM-EDS measurements.

[1] Ln stands for lanthanide (Eu, Tb, and La) dopant.

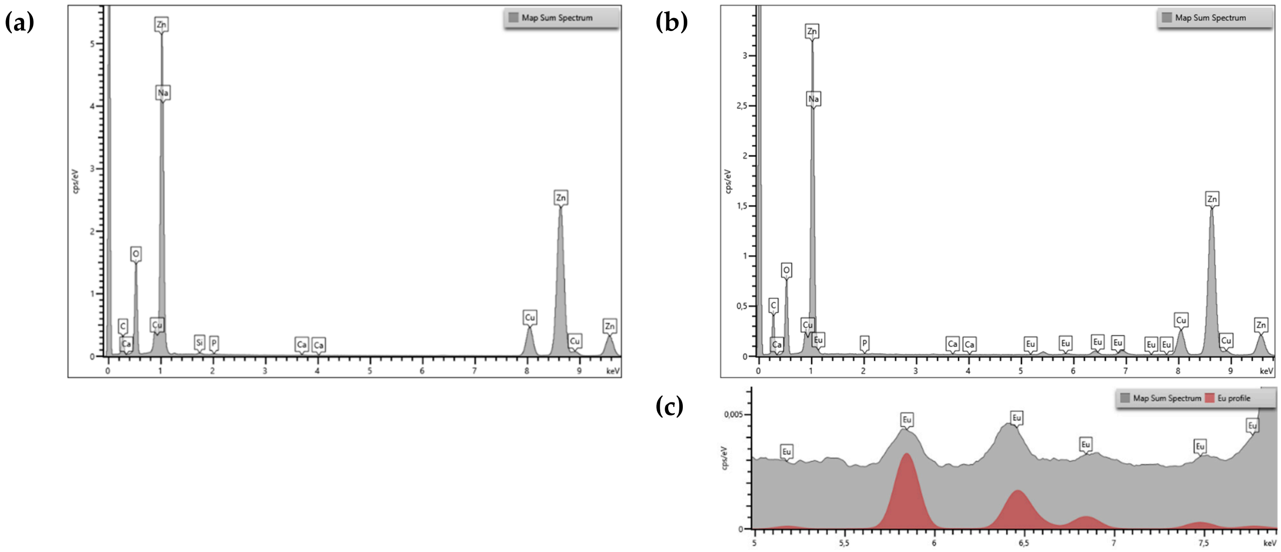

Figure S4. EDS map sum spectrum of ZnO (**a**) and ZnO:Eu (**b**). (**c**) Insets of (b) magnified in the 4.6–8.3 eV region, shows peak shapes of Eu L line series fitted to the spectrum by the Filtered Least Squares approach. EDS spectrum images (SIs) were acquired with 199 x 166-pixel dimensions and 62.4 ms dwell-time.

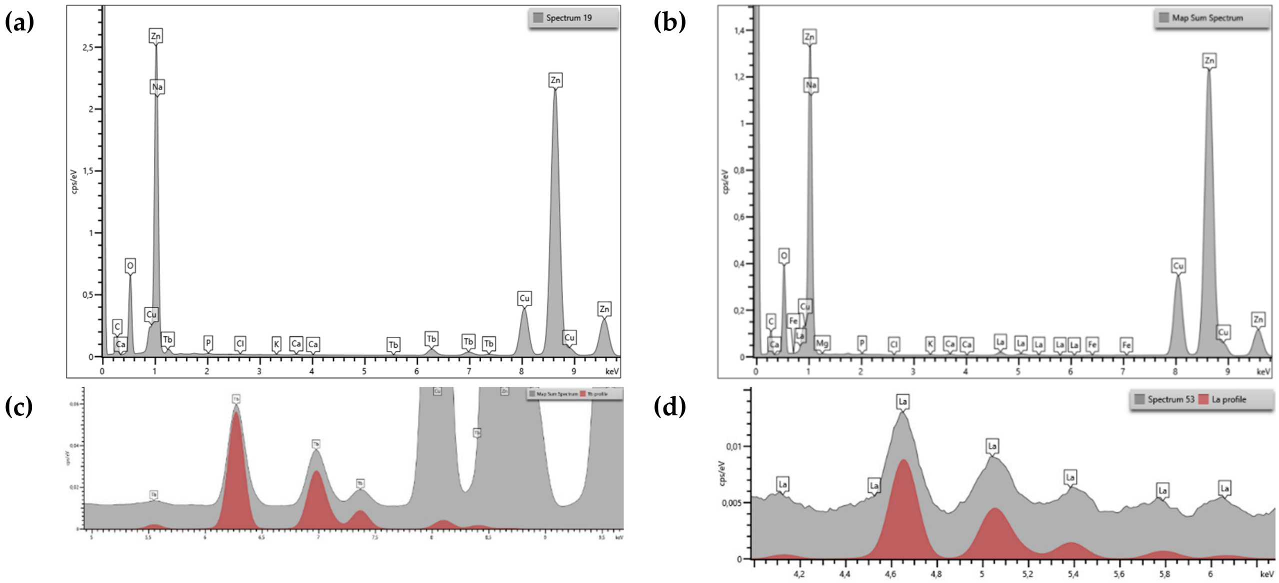

Figure S5. EDS map sum spectrum of ZnO:Tb (**a**) and ZnO:La (**b**). (**c**) Inset of (a) magnified in the 5.0–9.5 eV region, shows peak shapes of Tb L line series fitted to the spectrum by the Filtered Least Squares approach. EDS SIs were acquired with 1024 x 1024-pixel dimensions and 35 µs dwell-time. (**d**) Insets of (**b**) magnified in the 4.0–6.2 eV region, shows peak shapes of La L line series fitted to the spectrum by the Filtered Least Squares approach. EDS SIs were acquired with 512-pixel x 512-pixel dimensions and 50 µs dwell-time.

2. X-ray photoelectron spectroscopy (XPS)

The modified Auger parameter (α') values were calculated using the uncorrected Zn 2p photoelectron and $L_3M_{45}M_{45}$ Auger lines of Zn and compared to the literature value of 2010.4 eV for zinc oxide [1, 2].

$$\alpha' = E_\mathrm{b}(\mathrm{Zn2p_{3/2}}) + E_\mathrm{k}(Zn\mathrm{L_3M_{45}M_{45}})$$

$$\alpha' = E_\mathrm{b}(\mathrm{Zn2p_{3/2}}) + h\nu - E_\mathrm{b}(\mathrm{ZnL_3M_{45}M_{45}})$$

where E_b and E_k are the binding and kinetic energies of the corresponding photoelectron and Auger lines, respectively.

The obtained α' values of 2009.9 eV (ZnO, ZnO:Eu and ZnO:Tb) and 2010.0 eV (ZnO:La) confirmed the formation of ZnO. Zn 2p core-level XPS were determined.

Table S2. Binding energies of Zn $2p_{3/2}$, O 1s, O 1s (Zn-OH), C 1s (C-C/C-H), Na 1s, Ca $2p_{3/2}$, K $2p_{3/2}$, and Cl $2p_{3/2}$. [1] Ln stands for lanthanide (Eu, Tb, and La) dopant.

	Binding energy of photoemission lines (eV)					Elemental composition (at%)			
**Sample**	**Zn $2p_{3/2}$**	**O 1s**	**O 1s (Zn-OH)**	**Ln[1] 3d**	**C 1s (C-C/C-H)**	**Na 1s**	**Ca $2p_{3/2}$**	**K $2p_{3/2}$**	**Cl $2p_{3/2}$**
**ZnO**	2009.9	530.23	531.71	-	284.81	1071.84	347.33	296.44	198.89
**ZnO:Eu**	2009.9	530.18	531.66	1134.30<br>1164.40	284.80	1071.64	347.18	296.06	198.77
**ZnO:Tb**	2009.9	530.17	531.62	1239.97<br>1276.70	284.80	1071.60	347.20	296.10	198.72
**ZnO:La**	2010.0	530.14	531.61	835.06<br>851.8	284.78	1071.61	347.32	296.20	198.75

2.1. Core-level XPS from additional non-intentional dopants: Na 1s, K 2p, Ca 2p, and Cl 2p (all samples)

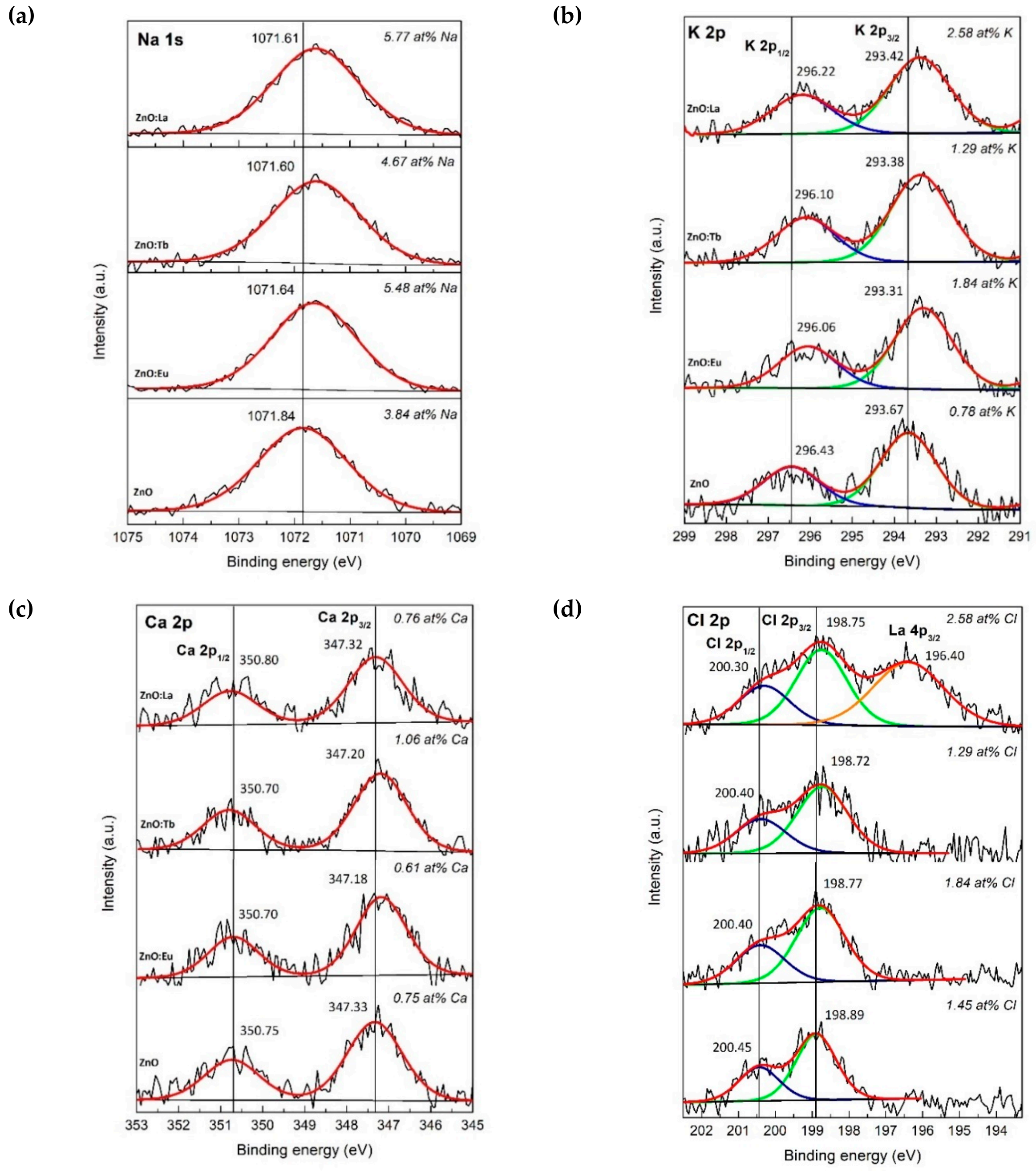

Figure S6. HRXPS spectra of (**a**) Na 1s; (**b**) K $2p_{3/2}$; (**c**) Ca $2p_{3/2}$ (**c**); and (**d**) Cl $2p_{3/2}$ core-level photoelectron lines from ZnO, and Eu, Tb, La doped ZnO samples.

The HRXPS spectra of Na 1s core-level exhibited in Fig. S6a show that at increasing concentrations of Na the binding energy shifts towards lower energies. Similarly, the spectra of K 2p (Fig. S6b) follow the same trend of lower binding energies with increasing K concentrations.

HRXPS spectra of Ca 2p (Fig. S6c), exhibit constant binding energies apparently not related to the concentration. The HRXPS spectra of Cl 2p (Fig. S6d), similarly to Ca 2p, show constant binding energies not influenced by the concentration of Cl in the sample.

The spectrum from the sample ZnO:La exhibits an additional photoemission line from La $4p_{3/2}$ centered at 196.4 eV.

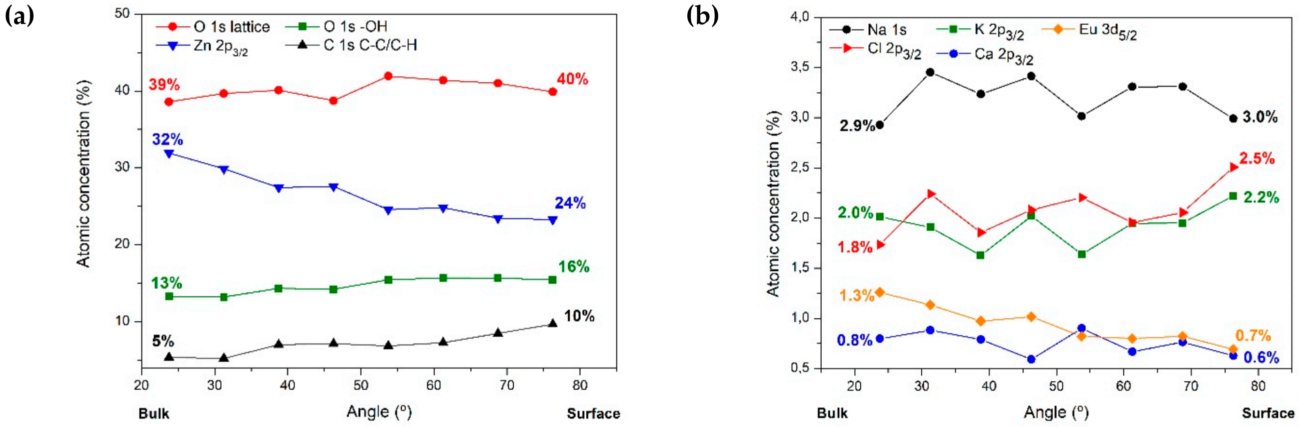

Figure S7. (**a**) ARXPS of Zn 2p, O 1s lattice, O 1s –OH and adventitious C 1s. (**b**) ARXPS (right) Na 1s, K $2p_{3/2}$, Cl $2p_{3/2}$, Ca $2p_{3/2}$, and Eu $3d_{5/2}$, from sample ZnO:Eu.

3. X-ray powder diffraction (XRPD)

Table S3. Selected bond distances and angles of ZnO, ZnO:Eu, ZnO:Tb, and ZnO:La.

	**ZnO**	**ZnO:Eu**	**ZnO:Tb**	**ZnO:La**
**Selected bond distances (Å)**				
Zn1-O1 (O in *c*-axis)	2.0150	2.0171 (16)	1.9931	1.9846 (0)
Zn1-O1′ (O off *c*-axis)	1.9661	1.9656 (5)	1.9727	1.9756 (0)
**Selected bond angles (°)**				
α ($\widehat{0Zn0'}$)	107.392	107.333 (42)	107.988	108.224 (0)
β ($\widehat{0'Zn0'}$)	111.467	111.523 (43)	110.913	110.690 (0)

Table S4. Domain size and average maximum strain of ZnO, ZnO:Eu, ZnO:Tb, and ZnO:La samples.

	**ZnO**	**ZnO:Eu**	**ZnO:Tb**	**ZnO:La**

**Microstructural analysis**				
**Average apparent size (Å)**	401.57 (33)	433.02 (12)	408.36 (33)	412.10 (33)
**Average maximum strain (%)**	13.9618 (207)	14.4734 (141)	11.3448 (146)	11.4910 (141)

4. Photoluminescence spectroscopy

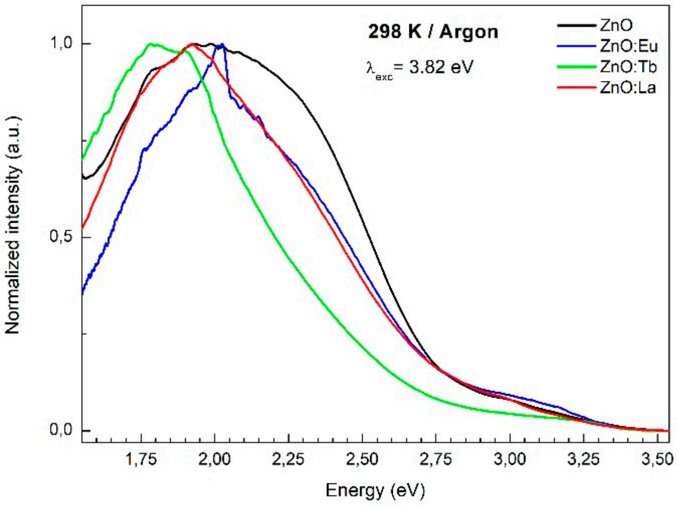

Figure S8. PL emission spectra performed at 298 K of ZnO, ZnO:Eu, ZnO:Tb, and ZnO:La NPs.

(a)

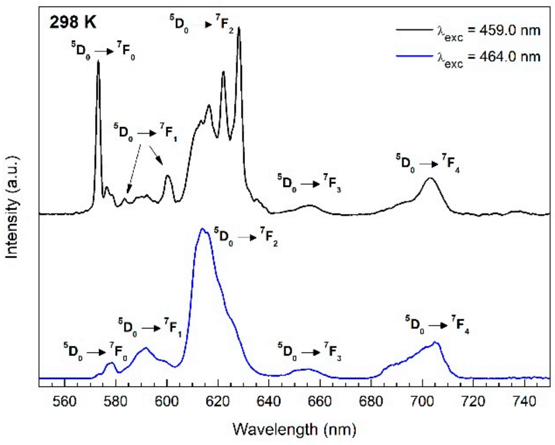

(b)

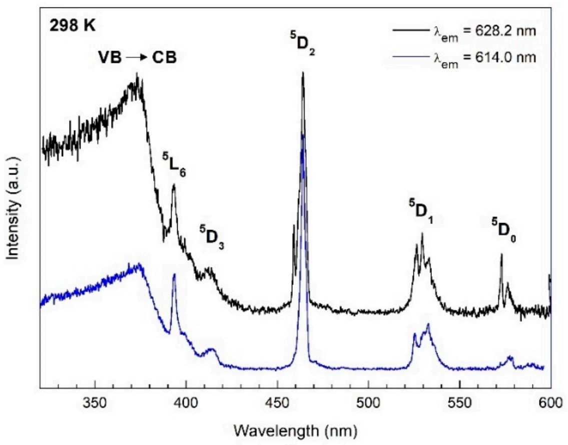

Figure S9. (**a**) Site-selective emission spectra of Eu^{3+} ions in ZnO:Eu at 298. (**b**) Site-selective excitation spectra of Eu^{3+} ions in ZnO:Eu at 298 K.

The relative quantum yields (QYs) were measured using a solution of Rhodamine 6G in ethanol (QY = 95%) as a reference material according to literature [3]. Quantum yields < 1% for all samples when dispersed in ethanol and excited at 325 nm were measured.

5. Calculation of average wt% values and average standard deviations

Quantitative EDS microanalysis in Aztec v4.3, displays elemental composition values in mass percent composition (wt%) with one standard deviation (σ), along with atomic percent composition (at%) values, based on counting statistics.

The calculation of an average wt% value for each element from different wt% values observed in different nanoparticles within a common sample, was realized by using an arithmetic mean equation:

$$\bar{x} = \frac{1}{n}\sum_{i=1}^{n} x$$

Where, x is the wt% value of each nanoparticle and n is the number of wt% values.

The calculation of the standard deviation for the previous average wt% value based on different sets of data, is not as trivial as the previous operation. The reason is because each individual wt% value given by Aztec software is in reallity a mean value itself given with one standard deviation and calculated from n observed wt% values. Therefore, to calculate the standard deviation for the average wt% value, one must know all n observed wt% values from all datasets and apply the following standard deviation equation:

$$\sigma = \sqrt{\frac{1}{kn}\sum_{1}^{kn}(x - \bar{x})^2}$$

where kn is the total number of values from k datasets, x is each observed wt% value and $\bar{x}$ is the average wt% value calculated from k datasets. The problem arises when we do not know all n observed wt% values (x), but only the *mean* wt% value itself and the standard deviation σ from each dataset. The solution to this problem is found when we acknowledge this calculation can be performed as an intraclass correlation, a model part of the Analysis of Variance (ANOVA) methodology developed by the statistician Ronald Fisher [4]. The intraclass correlation model is based on the treatment of all data points as they were from the same dataset having the same mean and standard deviation. Therefore, the standard deviation for the average wt% can be calculated from $\sigma^2 \cdot kn$ using the following equation:

$$\sigma^2 \cdot kn = \sum_{1}^{kn}(x-\bar{x})^2 = k\sum_{1}^{n\prime}(\bar{x}_p-\bar{x})^2 + \sum_{1}^{k}\sum_{1}^{n}(x-\bar{x}_p)^2$$

Where the first term is the sum of squares of the kn deviations of all the observations from the general mean (average wt%), the second is k times the sum of the squares of the n' deviations of the mean of each family from the general mean, and the third term is the summatory for k families of the sum of the n squares of the deviations of each individual measurement from the mean of the family to which it belongs. Knowing for each dataset (for each k) the number of counts in the spectrum (n), then kn is the sum of the counts from all datasets. Therefore, the variance for each dataset, $(\sigma')^2$, multiplied by the number of counts of its spectrum, it is equal to the third term in the previous equation.

$$(\sigma')^2 \cdot n = \sum_{1}^{n}(x-\bar{x}_p)^2$$

The average standard deviation for the average wt% value is:

$$\sigma = \left[\frac{1}{kn}\left(kk\sum_{1}^{n\prime}(\bar{x}_p-\bar{x})^2 + \sum_{1}^{k}\sum_{1}^{n}(x-\bar{x}_p)^2\right)\right]^{1/2}$$

where, $\bar{x}_p$ is the wt% value of each element in a dataset, and $\bar{x}$ is the average wt% value calculated from k datasets.

References

[1] Biesinger, M.C.; Lau, L.W.M; Gerson, A.R.; Smart, R.St.C. Resolving surface chemical states in XPS analysis of first row transition metals, oxides and hydroxides: Sc, Ti, V, Cu and Zn. *App. Surf. Sci.* **2010**, *257*, 887–898, doi:10.1016/j.apsusc.2010.07.086.

[2] Correia, F.C.; Bundaleski, N.; Teodoro, O.M.N.D.; Correia, M.R.; Rebouta, L.; Mendes, A.; Tavares, C.J. XPS analysis of ZnO:Ga films deposited by magnetron sputtering: Substrate bias effect. *App. Surf. Sci.* **2018**, *458*, 1043–1049, doi:10.1016/j.apsusc.2018.07.135.

[3] Xiong, H.M.; Liu, D.P.; Xia, Y.Y.; Chen, J.S. Polyether-Grafted ZnO Nanoparticles with Tunable and Stable Photoluminescence at Room Temperature. *Chem. Mater*. **2005**, *17*, 3062–3064, doi.org/10.1021/cm050556r.

[4] Fisher, R.A. In *Statistical methods for research workers*, 5th ed.; Crew, F.A.E., Cutler, D.W., Eds.; Oliver and Boyd: Edinburgh, UK, 1946; pp. 210–214.
